# Duodenal duplication cyst at the second part of the duodenum with congenital duodenal position anomaly completely resected by laparoscopic partial duodenectomy: a case report

**DOI:** 10.1186/s40792-024-01875-0

**Published:** 2024-03-29

**Authors:** Yoichi Nakagawa, Hiroo Uchida, Satoshi Makita, Takahisa Tainaka, Akinari Hinoki, Chiyoe Shirota, Wataru Sumida, Hizuru Amano, Akihiro Yasui, Yoko Kano, Takuya Maeda, Daiki Kato, Yousuke Gohda

**Affiliations:** 1 Department of Pediatric Surgery, Nagoya University Graduate School of Medicine, 65 Tsurumai-Cho, Showa-Ku, Nagoya, Aichi 466-8550 Japan; 2 Department of Rare/Intractable Cancer Analysis Research, Nagoya University Graduate School of Medicine, 65 Tsurumai-Cho, Showa-Ku, Nagoya, 466-8550 Japan

**Keywords:** Congenital duodenal position anomaly, Duodenum, Duplication of the alimentary tract, Laparoscopy

## Abstract

**Background:**

Duodenal duplication cysts (DDC) are rare duplications of the alimentary tract. Their treatment depends on their size and location. A radical treatment is total resection, if possible. However, partial excision, puncture, and marsupialization can be selected to prevent surgical injury to the pancreaticobiliary tract despite the risk of recurrence. There are some reports of pancreaticoduodenectomy for DDC because of the risk of recurrent symptoms and malignancy. However, this is considered excessively invasive for DDC, particularly in pediatric cases, because of its extremely low rate of malignancy and high morbidity and mortality rates. We encountered a case of DDC with a congenital duodenal position anomaly occurring in the second part of the duodenum. Taking advantage of the congenital duodenal position anomaly, the DDC was completely resected without injuring the pancreaticobiliary duct.

**Case presentation:**

A 6-year-old boy was diagnosed with a duodenal duplication cyst with obstruction. There was a congenital duodenal position anomaly. The distal second part of the duodenum was the dorsal side of the proximal second part of the duodenum and ascended upward from the proximal second part of the duodenum. The third and fourth parts of the duodenum ran downward to the left and posterior parts of the portal vein, forming the ligament of Treitz. Complete laparoscopic resection of the duodenal duplication cyst and the second to fourth parts of the duodenum, and duodenojejunostomy with retrocolic reconstruction was performed because the duodenum was easily mobilized to the ligament of Treitz owing to the duodenal position anomaly. The duodenojejunostomy with retrocolic reconstruction achieved a more physiologically normal appearance compared to what would have been achieved with a Roux-en-Y reconstruction. The patient was discharged on postoperative day 12 without any complications.

**Conclusions:**

The procedure used in this case might not be easily applied in all laparoscopy cases. However, it could be an option for duodenal duplication cysts with congenital duodenal position anomalies.

## Background

Alimentary tract duplication is a rare congenital anomaly characterized by an intimate attachment to the native gastrointestinal tract, smooth muscle coat, and alimentary mucosal lining [1]. Duodenal duplication cysts (DDCs) are rare and account for only 4% of all alimentary tract duplications [2]. Treatment of DDCs depends on the size and location of the duplicates. Total resection is the radical treatment, if possible. However, despite the high risk of recurrence, partial excision, puncture, and marsupialization can be selected to prevent surgical injury to the pancreaticobiliary tract. There are some reports of pancreaticoduodenectomy for DDCs because of the risk of recurrent symptoms and malignancy. There has been a small number of reports on malignant DDCs in adult cases—although a DDC is a potentially malignant disease [3]—and no malignant cases in children [4]. In pediatric, adolescent, and young adult patients undergoing pancreatoduodenectomy, in-hospital mortality was 1%, new-onset diabetes mellitus occurred in 8% of the cases, and exocrine pancreatic insufficiency developed in 27% of the cases [5]. Considering the above, pancreatoduodenectomy is too invasive for DDCs, particularly in pediatric cases. We encountered a case of DDC with a congenital duodenal position anomaly occurring in the second part of the duodenum. Taking advantage of the duodenal position anomaly, the DDC was completely resected without injuring the pancreaticobiliary duct. Herein, we present our case and discuss the surgical procedure.

## Case presentation

**Fig. 1:**
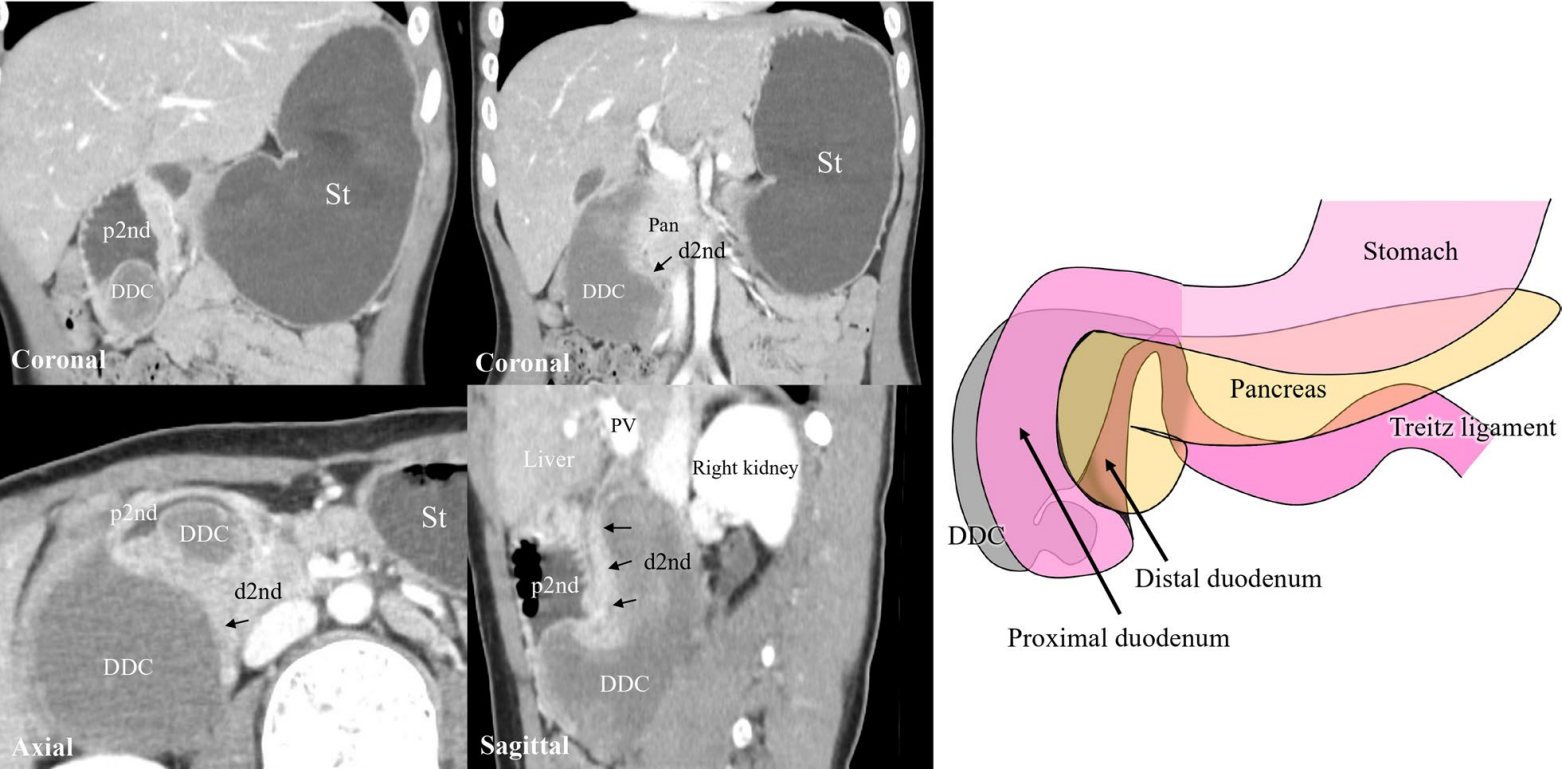
Computed tomography. DDCs are present proximal to the second distal part of the duodenum and cause obstruction, indicating an intra-cystic hemorrhage. The schema shows the DDC location. *DDC* duodenal duplication cyst, *St* stomach, *p2nd* proximal second part of the duodenum, *d2nd* distal second part of the duodenum, *pan* pancreas, *PV* portal vein

**Fig. 2:**
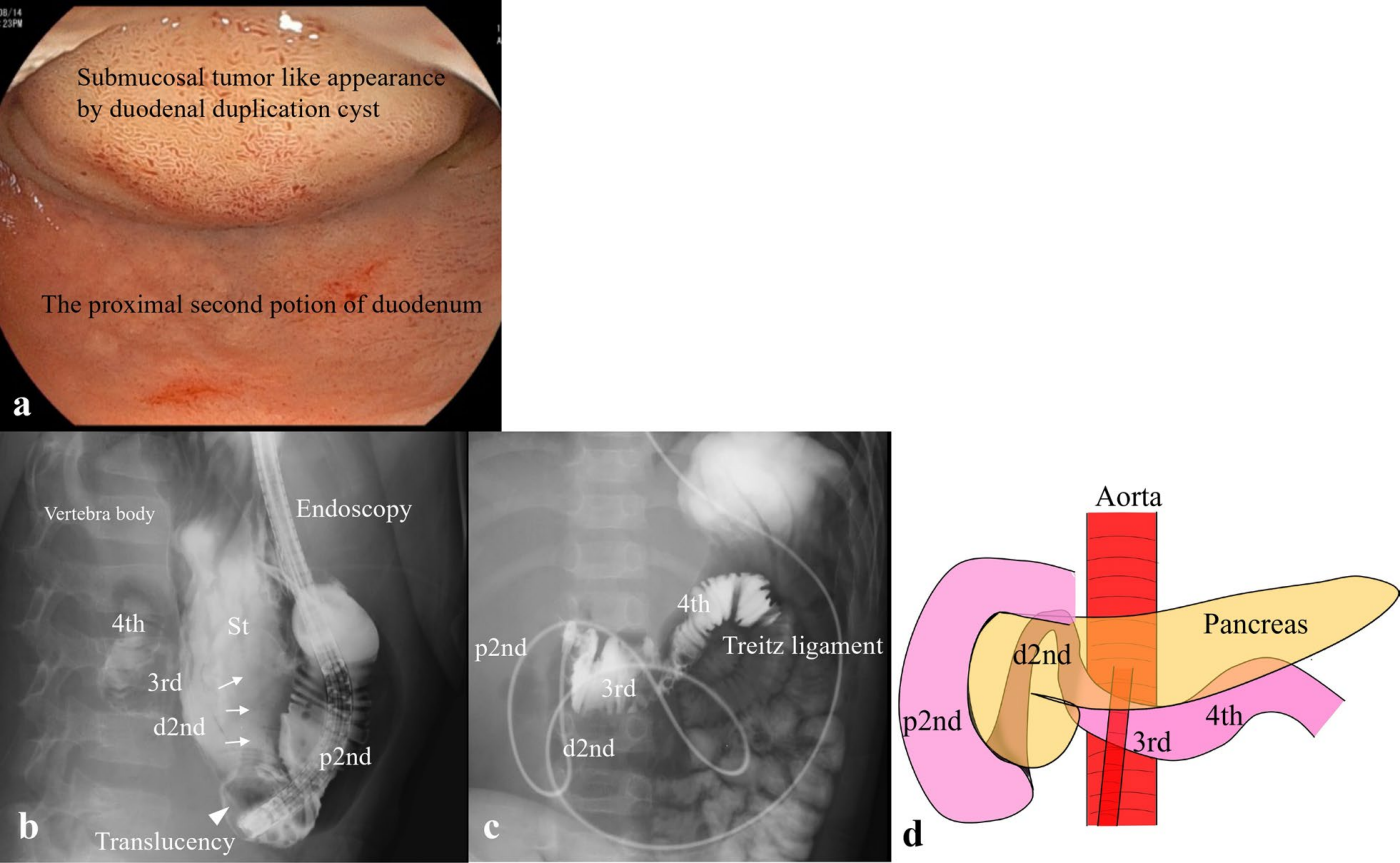
Upper endoscopy findings. **a** A duodenal duplication cyst compresses the second part of the duodenum. **b**, **c** Contrast examination during endoscopy shows translucency in the second part of the duodenum and stenosis of the distal second part of the duodenum (white arrow). **d** The schema shows a congenital duodenal position anomaly. *St* stomach, *p2nd* proximal second part of the duodenum, *d2nd* distal second part of the duodenum, *3rd* third part of the duodenum, *4th* fourth part of the duodenum

A 6-year-old Japanese boy with no relevant medical history presented with vomiting. Laboratory data revealed hypochloremic alkalemia with dehydration. Computed tomography revealed a congenital duodenal position anomaly and DDC in the second part of the duodenum (Fig. 1). The DDC was identified as internal bleeding, but there was no air, gas, or apparent communication within the duodenum (Fig. 1). Duodenal obstruction due to DDC was diagnosed. The cause of the duodenal obstruction was considered to be the internal bleeding of the DDC because the patient had no apparent history of obstructive symptoms such as abdominal pain and vomiting. Upper endoscopy showed that the second part of the duodenum was completely obstructed by the DDC (Fig. 2a), and the location of the papilla of Vater was at the proximal site of the obstruction. Upper gastrointestinal fluoroscopy showed a congenital duodenal position anomaly (Fig. 2b–d). An elemental diet tube was placed under endoscopy, and enteral nutrition was initiated. After correcting electrolyte imbalance and dehydration, the patient underwent laparoscopic surgery. At first, we planned for DDC resection with mucosal stripping with or without a Roux-en-Y duodenojejunostomy reconstruction, considering the large defect hole of the DDC.

**Fig. 3:**
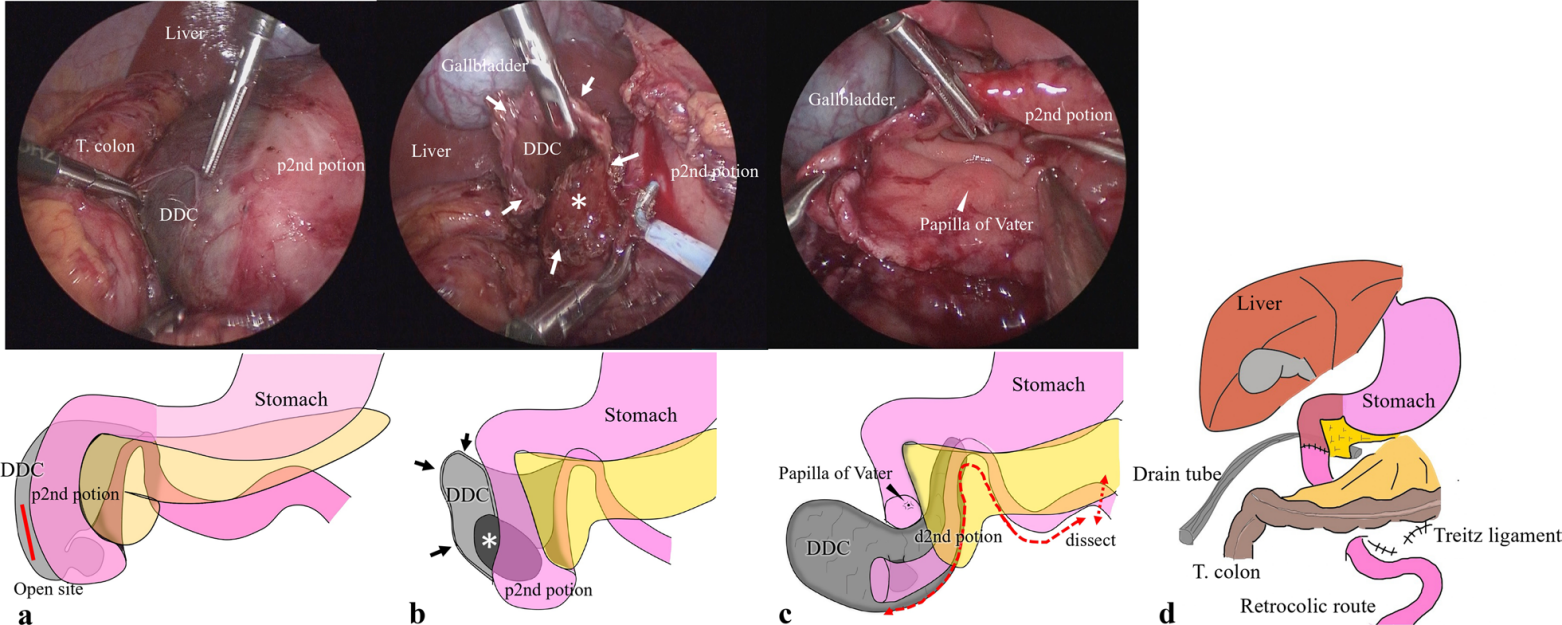
Operative findings and schema. **a** The DDC contains blood and compresses the second part of the duodenum from the dorsal side. **b** The DDC is opened to check the cyst lumen (white arrow). The intestinal mucosa lines the lumen, and a protruding cyst is confirmed at the distal second part of the duodenum (*). **c** The second part of the duodenum is resected. The papilla of Vater is indicated with a white arrowhead. **d** The schema shows the resected duodenum location and partial duodenectomy with retrocolic duodenojejunostomy. *p2nd* proximal second portion of the duodenum, *d2nd* distal second portion of the duodenum

An umbilical Benz incision was made. A multichannel access port with two 5-mm trocars was also inserted through the incision. Subsequently, two 5-mm trocars and a 3-mm trocar were inserted into the right and left flank abdomen and epigastrium. First, Kocherization was performed to mobilize the duodenum. The DDC was opened for decompression and was found to contain old blood (Fig. 3a). The DDC originated from the distal second part of the duodenum; the distal second part was dorsal to the proximal second part of the duodenum, ascending upward from the proximal second part of the duodenum. The third and fourth parts of the duodenum ran downward to the left and posterior parts of the portal vein, forming the ligament of Treitz. The DDC lumen and protruding region into the second part of the duodenum were confirmed (Fig. 3b). The second part of the duodenum, on the opposite side of the pancreatic head, was opened to check for the protruding part of the DDC. The protruding part of the DDC was present distal to the papilla of Vater (Fig. 3c). While checking the intraluminal space of the DDC, the distal second to fourth parts of the duodenum were easily dissected from the surrounding tissue and ligament of Treitz. Hence, complete laparoscopic resection of the DDC and the second to fourth parts of the duodenum, and duodenojejunostomy with retrocolic reconstruction were performed.

**Fig. 4:**
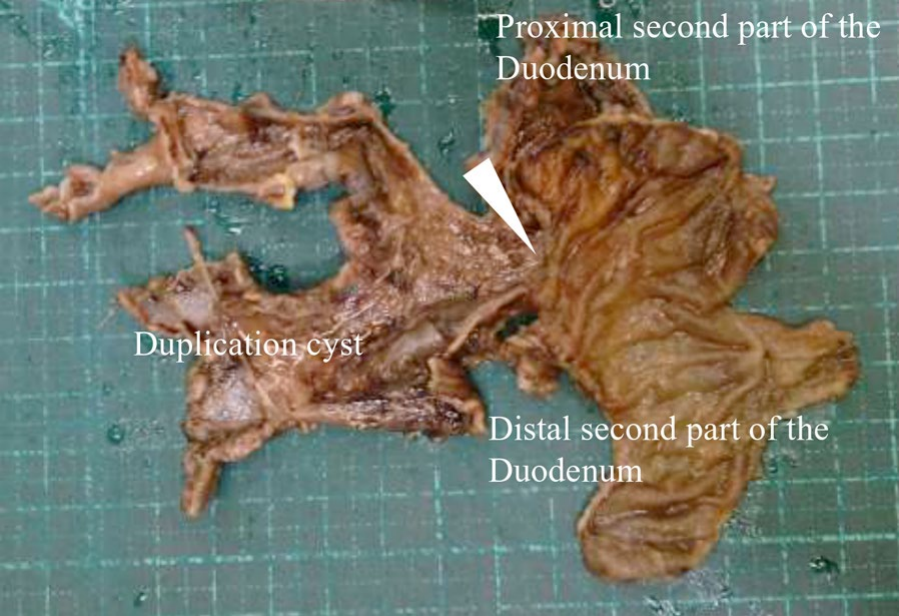
Macroscopic examination of the DDC. The DDC was in communication with the duodenum at the proximal second part of the duodenum (white arrowhead)

The partial duodenectomy, involving the distal second to fourth parts of the duodenum, was performed laparoscopically, followed by extracorporeal retrocolic reconstruction. Then, the hole at the ligament of Treitz and Petersen’s defect was closed laparoscopically using 4-0 absorbable sutures. The remaining second part of the duodenum with the DDC was fully resected distal to the papilla of Vater. Finally, duodenojejunostomy was performed with single-layer end-to-end anastomosis using 4-0 absorbable sutures. The surgical procedure is described in the schema provided in Fig. 3d. The postoperative course was uneventful. The patient was discharged on postoperative day 12. Macroscopic examinations showed that the DDC communicated with the duodenum at the proximal second part of the duodenum (Fig. 4). Microscopic examination showed that the DDC was partly lined by duodenal mucosa. However, the ectopic mucosa and the common muscle layer of the duodenum could not be evaluated because the hemorrhage caused granulation formation, fibrogenesis, and cystic region between the submucosa and muscularis propria. However, comprehensively, these findings were consistent with communicating type DDC. The patient remains under follow-up and 4 months after surgery he exhibits no symptoms. Long-term follow-up is planned.

## Discussion

The best treatment for DDC is complete resection to prevent future complications, including malignancy, because DDC may contain dysplasia mucosa or early malignant tissue. Three cases of malignancy arising from DDC were reported [6]. However, excision of the DDC, as much as possible, and mucosal stripping were selected when complete resection was hindered by the site and size of the DDC. Theoretically, pancreatoduodenectomy completely resects DDC wherever it is present but should remain the ultimate option [7] for long-term quality of life.

The preoperative examination revealed that the DDC was relatively large and intimately attached to the second part of the duodenum. As previously reported, subtotal resection of the DDC and mucosal stripping were considered to be a valid treatment option. Endoscopy-assisted windowing [8] is another option in unresectable cases, although maintaining the mucosal layer bears a slight risk of malignancies [6]. Mucosal stripping is safe and has few complications. However, postoperative stenosis or stricture could occur. Duodenal lesions > 30 mm carry a risk of postoperative complications following endoscopic mucosal resection; duodenal stricture occurred in 0.8% of cases [9]. Considering that our patient still has a long life and that there would have been the possibility of future complications such as stenosis, we considered that complete resection with reconstruction was the best method to treat the DDC ensuring no future complications. Endoscopy-assisted laparoscopic full-thickness resection could have been another option [10], if an endoscope could have been inserted.

We preoperatively planned to perform total resection with a defect hole anastomosed to the jejunum with Roux-en-Y reconstruction rather than simple suture closure to prevent postoperative duodenal stricture. Intraoperative laparoscopic observation showed that the papilla of Vater was distant from the DDC. Furthermore, the duodenum was easily mobilized to the ligament of Treitz because of a congenital duodenal position anomaly. Hence, total resection of the DDC with the second to fourth parts of the duodenum via a retrocolic route duodenojejunostomy was performed. We selected this reconstruction approach because it guarantees a more physiologically normal food pathway, whereas a Roux-en-Y anastomosis could cause Roux stasis syndrome [11–13].

To our knowledge, the same duodenal position anomalies have been reported in three cases [14–16]. Pham et al. indicated that this anomaly was caused by folding of the second part of the duodenum at the junction of the foregut, midgut, or proximal midgut [15]. They also reported that the second and third parts of the duodenum did not adhere strongly to the adjacent organs. Hence, duodenal mobilization was not difficult [15]. In the present case, the mobilization of the duodenum was facilitated using laparoscopy. The procedure used in this case was not applied as easily in all previously reported cases by laparoscopy because in most cases the second to fourth parts of the duodenum were firmly fixed with the ligament of Treitz. Despite the relatively low occurrence rate, DDC is related to malignancies, and complete resection is an ideal treatment option when possible. The procedure reported herein may be a valid option for large DDCs distal to the Vater papilla, particularly in cases of a congenital duodenal position anomaly.

## Conclusions

We successfully performed a complete laparoscopic resection of a DDC. Although partial resection of the DDC with mucosal stripping with Roux-en-Y reconstruction is an alternative treatment option, complete resection of the DDC with the second to fourth parts of the duodenum was selected during surgery because the duodenum was easily mobilized to the ligament of Treitz owing to a congenital duodenal position anomaly. In addition, duodenojejunostomy with retrocolic reconstruction ensured a more normal physiology compared to Roux-en-Y reconstruction.

## Data Availability

Not applicable.

## Abbreviation

DDC: Duodenal duplication cyst
